# Arginine 37 of Glycine Linker Dictates Regulatory Function of HapR

**DOI:** 10.3389/fmicb.2020.01949

**Published:** 2020-08-21

**Authors:** Manjula Ekka, Abhisek Mondal, Richa Singh, Himanshu Sen, Saumen Datta, Saumya Raychaudhuri

**Affiliations:** ^1^Council of Scientific and Industrial Research (CSIR), Institute of Microbial Technology, Chandigarh, India; ^2^Council of Scientific and Industrial Research (CSIR), Indian Institute of Chemical Biology, Kolkata, India

**Keywords:** HapR, *Vibrio cholerae*, quorum sensing, linker, arginine, DNA binding, molecular dynamics

## Abstract

HapR is designated as a high cell density quorum sensing master regulatory protein of *Vibrio cholerae*. It is a member of the TetR family protein and functions both as an activator and a repressor by directly communicating with cognate promoters, thus controlling the expression of a plethora of genes in a density-dependent manner. Molecular insights reveal the domain architecture and further unveil the significance of a cross talk between the DNA binding domain and the dimerization domain for the functionality of the wild-type protein. The DNA binding domain is made up of three α-helices, where a helix-turn-helix motif spans between the helices α2 and α3. The essentiality of the glycine-rich linker linking helices α1 and α2 came into prominence while unraveling the molecular basis of a natural non-functional variant of HapR. Subsequently, the importance of linker length was demonstrated. The present study, involving a series of biochemical analyses coupled with molecular dynamics simulation, has illustrated the indispensability of a critical arginine within the linker at position 37 contributing to HapR–DNA binding activity.

## Introduction

Communication is an essential characteristic of life. Even microbes, once perceived as organisms that maintain a reclusive lifestyle, also participate in intra- and interspecies social networking to shape their behavior in diverse environmental settings. Such communication of the microbial world known as “quorum-sensing” has become a major focus of research. Currently, a large body of cumulative knowledge strongly underpins how the quorum sensing communication process allows microbes to coordinate a plethora of cellular events including pathogenesis and survival in various ecological niches (Velicer, 2003).

*Vibrio cholerae* causes cholera, a life-threatening diarrheal disease with worldwide distribution (Camacho et al., 2018). In recent past, an elaborate study on the quorum sensing of *V. cholerae* unveiled its impact on the individual and collective behavior of this bacterium. Acting in concert, a myriad of factors including small metabolites, non-coding RNAs, and regulatory proteins control the optimal performance of the quorum sensing sensory network in *V. cholerae* (Lenz et al., 2004; Papenfort et al., 2017). Among the various regulatory proteins, HapR is crowned as the “high cell density master regulator.” Functionally, HapR belongs to the TetR family regulatory proteins, and it modulates a large number of disparate physiological events, thus governing virulence to environmental survival of the bacterium (Ng and Bassler, 2009). Crystal structure analysis reveals a two-domain architecture where the N-terminal DNA binding domain comprised three α-helices and the remaining six α-helices are found in the C-terminal-located dimerization domain (De Silva et al., 2007). Mechanistically, HapR communicates with cognate promoters by recognizing unique DNA binding motifs (Tsou et al., 2009), thereby fulfilling its commitment as a high cell density master regulator by regulating the constellation of diverse cellular activities. The motif 1 sequence comprised a dyad symmetry and a consensus “AATAR” (where R represents A or G). On the contrary, the motif 2 sequence is devoid of any dyad symmetry, but includes highly conserved consensus “TGT” (Tsou et al., 2009). Besides HapR, the crystal structure of SmcR, a HapR homolog of *Vibrio vulnificus*, has also revealed a similar architecture, having an N-terminal DNA binding domain and a C-terminal dimerization domain. Like HapR, SmcR also interacts with many promoters (Kim et al., 2010).

Previously, we have shown the contribution of a conserved glycine 39 located in the DNA binding domain in HapR function (Dongre et al., 2011). It is to be noted that all residues in the glycine linker are highly conserved in HapR homologs. In a continuing effort, we have further established that the length of this glycine-rich linker is also important for HapR function as a DNA binding protein (Singh et al., 2014). Recognition that the linker composition is also linked to protein function (Van Leeuwen et al., 1997; Seedorff and Schleif, 2011) further prompted us to embark on the present work.

Our present work focuses on the contribution of each residue of the glycine-rich linker in the DNA binding activity of HapR. Combined *in vitro* and *in vivo* results clearly identified a critical arginine at position 37 (R^37^) imparting in the HapR-mediated cross talk with various cognate promoters. Further molecular dynamics studies suggested the role of arginine in positioning the DNA binding domain and facilitating the interaction with cognate DNA.

## Materials and Methods

### Bacterial Strains and Media

The bacterial strains and plasmids used in this study are listed in Supplementary Table 1. *V. cholerae* strains were derived from a non-O1, non-O139 strain S7, and serogroup O37. In addition, *V. cholerae* strains V2_S_ and GK178 were also included in this study. The strains were maintained at −70°C in Luria–Bertani (LB) medium containing 20% glycerol. *Escherichia coli* BL21 (DE3, Novagen) was used for the overexpression of proteins. All strains were propagated at 37°C in liquid with agitation or on solid (1.5% agar) in Luria broth, unless mentioned otherwise. For protease assay, *V. cholerae* recombinant strains were grown with aeration at 37°C in tryptic soya broth without dextrose (TSB-D). The growth medium was supplemented with streptomycin (100 μg ml^–1^), chloramphenicol (17 μg ml^–1^), and ampicillin (100 μg ml^–1^), whenever appropriate. All antibiotics were purchased from Sigma-Aldrich. Media ingredients were purchased from Himedia and Difco.

### Site-Specific Mutagenesis

All the mutants were constructed using QuickChange PCR kit (Stratagene) as per the manufacturer’s guidelines. The primers are listed in Supplementary Table 2. Positive clones were sequenced in their entirety to confirm the clones and desired mutations at the corresponding positions. The desired constructs were further transformed into protease-negative strains of *V. cholerae* S7 and V2_*S*_.

### Protease Assay

Protease activity was measured by employing an azocasein assay described earlier (Raychaudhuri et al., 2006). Briefly, wild-type and recombinant derivatives of *V. cholerae* strains V2_S_ and S7 (Supplementary Table 1) were grown in TSB-D, containing appropriate antibiotics, with agitation to stationary phase at 37°C. Of the stationary phase culture supernatant, 100 μl was incubated with 100 μl azocasein (5 mg ml^–1^ in 100 mM Tris, pH 8.0) for 1 h at 37°C. The reaction was stopped by the addition of 400 μl of 10% trichloroacetic acid (TCA). After centrifugation, the supernatant was transferred to 700 μl of 525 mM NaOH and the optical density was determined at 442 nm. One azocasein unit was defined as the amount of enzyme producing an increase of 0.01 optical density (OD) units per hour.

### Colony Morphology and Motility Assay

Colony corrugation was examined as per published protocols (Giglio et al., 2013). Briefly, overnight-grown cultures were diluted in fresh medium and grown to an OD_600_ = 0.3–0.4. Five microliters of the secondary culture was spotted on LB agar containing appropriate antibiotics. The plates were incubated at 30°C and photographs were taken after 48 h. For motility assays, all recombinant strains were grown overnight on LB agar, followed by the inoculation of a single colony by toothpick on plates composed of 1% tryptone, 0.5% NaCl, and 0.3% agar (Moisi et al., 2009). The plates were incubated at 37°C and photographs were taken after 12 h.

### Hemagglutination Assay

Hemagglutinin assay was done as described elsewhere (Benitez et al., 2001). The cultures were grown overnight and the cells were pelleted to collect the supernatant. The chicken red blood cells collected from a slaughterhouse were washed twice with normal saline (0.85% NaCl) and once with Krebbs-Ringer-Tris (KRT) buffer (128 mM NaCl, 5.1 mM KCl, 1.34 mM MgSO_4_, 2.7 mM CaCl_2_, 10 mM Tris, pH 7.5). The red blood cells (RBCs) were resuspended in the KRT buffer at 1.2% (vol/vol). A serial dilution of 50 μl of the supernatant in the KRT buffer was mixed with 50 μl of freshly prepared chicken erythrocytes in the wells of a 96-well polystyrene V-bottom microtiter plate. The mixture was then incubated at room temperature for 30 min and the hemagglutination monitored visually.

### Protein Purification

Wild-type and mutant proteins were purified by Ni-NTA chromatography. The genes encoding these proteins were cloned into the *Nde*I-*Bam*HI site of the pET15b vector (Novagen) to generate N-terminal 6X His-HapR fusion protein. All positive clones were sequenced and subsequently transformed into *E. coli* BL21 (DE3). After induction with 0.4 mM isopropyl ß-D-1-thiogalactopyranoside (IPTG) at 37°C, HapR proteins were purified through Qiagen Ni^2+^-nitrilotriacetic acid columns. All proteins were dialyzed overnight in a solution of buffer A containing 10 mM Tris, pH 7.9, 300 mM KCl, and 0.1 mM EDTA.

### Electrophoretic Gel Mobility Shift Assay With Promoter Regions of *hapA*, *aphA*, and *vc0900*

Gel mobility shift assay was done essentially as described earlier (Dongre et al., 2011; Singh et al., 2013). Briefly, the promoter regions of *vca0865*(*hap*A), *vc2647*(*aph*A), and *vc0900*(*cdgG*), respectively, were amplified with primer pairs as listed in Supplementary Table 2 from the genomic DNA of *V. cholerae* strains V2 and S7. The fragments were gel purified and end labeled with γ-dATP^32^ using T4 polynucleotide kinase (New England Biolabs^®^_inc_). The binding reaction was carried out with 4 ng of labeled fragments in 10 mM Tris–HCl, pH 7.9, 1 mM EDTA, 1 mM dithiothreitol (DTT), 60 mM KCl, 10% glycerol, 5 μg bovine serum albumin (BSA), and 0.5 μg poly(dI-dC) in a 20 μl reaction volume for 20 min at 26°C. The reaction mixture was applied to a 5.5% native polyacrylamide gel and subjected to electrophoresis in 1 × TAE, pH 8.5, at 4°C. Gel electrophoresis was performed in a GIBCO-BRL gel apparatus (model V16-2). The gel was dried and autoradiographed to examine the shift of the band.

### Western Blot Analysis

To examine HapA production, Western blot analysis was carried out with 12 h grown spent cultures of recombinant strains harboring alanine variants along with the vector and wild-type controls. The cell-free supernatants were centrifuged and concentrated to 200 μl (10-fold) through 10 kDa Amicon ultracentrifugal filters (Millipore). The protein concentration was determined by the bicinchoninic acid (BCA) method and subjected to 12% sodium dodecyl sulfate polyacrylamide gel electrophoresis (SDS-PAGE). Protein samples were electrophoretically transferred onto an Immobilon-P polyvinylidene fluoride (PVDF) membrane (Millipore). The Hap protein was detected by probing rabbit anti-Hap serum primary antibody at a dilution of 1:2,500 and horseradish peroxidase (HRP)-conjugated goat anti-rabbit immunoglobulin G (IgG) secondary antibody at a dilution of 1:5,000. Molecular masses were deduced by referring to the protein molecular weight markers.

Western blot analysis was done to check the cellular stability of the wild-type and mutant proteins. Briefly, *V. cholerae* strain S7 harboring FLAG-tagged recombinant derivatives of the wild-type and mutant constructs were grown in tryptic soy broth (TSB) without glucose overnight with agitation. The protein samples were prepared from an equal number of bacterial cells and separated on 12% gel by SDS-PAGE. For immunoblotting, the proteins were transferred to Immobilon-P PVDF membrane (Millipore) at 75 mA for 1 h. The membrane was subsequently blocked in phosphate-buffered saline (PBS) with 5% skim milk at 37°C for 2 h and then shifted to 4°C overnight with shaking. Blot was then washed in PBS Tween (PBST) five times for 10 min each, incubated in monoclonal HRP-conjugated anti-flag (Sigma Aldrich) at a dilution of 1:5,000 in PBST with 2% skim milk for 1 h, and again washed in PBST five times for 10 min each. The proteins were visualized using the Luminata Forte Western HRP substrate (Millipore). Molecular masses were calculated with reference to the SDS-PAGE molecular mass standards (broad range) from NAX-GEN ALPHA PS ladder from Genetix Biotech.

### *In vivo* Oligomerization

The *V. cholerae* S7 strain bearing FLAG-tagged recombinant derivatives of the wild-type and mutant constructs were grown overnight. Equal numbers of cells were pelleted in duplicate for each culture and resuspended in protein sampling buffer ± DTT (100 mM) (Yang et al., 2013). The samples were then subjected to SDS-PAGE. Protein HapR was detected by Western blot using HRP-conjugated monoclonal anti-FLAG tag antibody (Sigma).

### β-Galactosidase Reporter Assay

The LacZ reporter assay was performed as reported earlier (Miller, 1972). For the β-galactosidase assay, *V. cholerae* strain GK178 (*aphA-lacZ* fusion) (Kovacikova and Skorupski, 2002) was used by propagating the cultures overnight and diluting in AKI medium (Iwanaga et al., 1986) at 1:100 dilution. The assay was carried out after harvesting the cells at 0.3–0.4 OD_60__0n__*m*_. The cell pellets were washed with Z-buffer (60 mM Na_2_HPO_4_⋅7H_2_O, 40 mM NaH_2_PO_4_×H_2_O, 10 mM KCl, 1 mM MgSO_4_⋅7H_2_O, and 50 mM β-mercaptoethanol) and resuspended in Sigma cell lytic buffer at a dilution of 1:10. The cells were sonicated at 20% amplitude for two cycles (10 s on and 10 s off). Of the clear lysate, 150 μl was mixed with 50 μl of *ortho*-nitrophenyl-β-galactoside [4 mg/ml in Z-buffer (pH 7) without β-mercaptoethanol] in 96-well plates and incubated at 30°C for 45 min. The β-galactosidase activity was observed by measuring the absorbance at 420 and 550 nm to calculate the Miller units (Miller, 1972).

### Molecular Dynamics Simulation

Modeling of the B-DNA was performed using 3D-DART (van Dijk and Bonvin, 2009) web server. Energy minimization of the DNA was performed using the steepest descent algorithm with AMBER ff99bsc0 force field (Wang et al., 2004). The DNA was docked into HapR using Genetic Algorithm as the search parameter in the AutoDock-4.2 package. The output of the docking run was generated with the Lamarckian GA algorithm in AutoDock (Morris et al., 2009). The docked model was parameterized using AMBER ff99bsc force field and also applied to the system. Particle mesh Ewald algorithm was used for long-range electrostatics calculations and a 1.4 nm cutoff was used for short-range non-bonded interaction calculations. Periodic boundary condition was also applied to the system. The structure was well equilibrated using a 3 nm cubic box with TIP3P water. NaCl (100 mM) was added to the system with neutralizing counterions. Energy minimization was performed using the steepest descent minimization algorithm and a two-phase equilibration was followed thereafter. In the first phase, the structure was equilibrated with 50 ns of constant volume (NVT) ensemble. In second phase, another 50 ns of constant pressure (NPT) equilibration was performed. After the equilibration cycles, production molecular dynamics (MD) was carried out for 80 ns with no position restraint applied to the protein–DNA complex. During production MD, a Nosé–Hoover thermostat (Nosé, 1984; Hoover, 1985) was used for maintaining temperature and Parrinello–Rahmanbarostat (Parrinello and Rahman, 1981) was used for isotropic regulation of pressure. All the simulations were performed using GROMACS-0.4.6.2 package (Hess et al., 2008). Trajectory analysis and visualizations were performed using Chimera (Pettersen et al., 2004).

## Results

### Arginine 37 of Glycine Linker Controls the DNA Binding Activity of HapR

**FIGURE 1:**
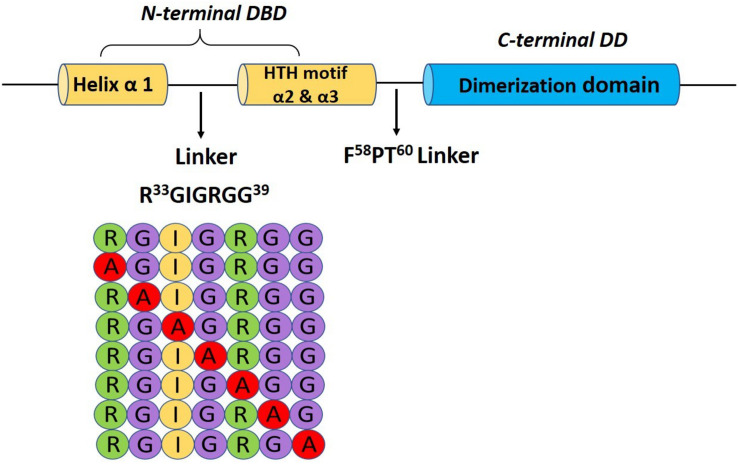
Pictorial representation of the glycine-rich linker (R^33^GIGRGG^39^) bridging the α-helix 1 and the HTH motif (α2 and α3) in the N-terminal binding domain and a schematic diagram showing the alanine scanning library.

**FIGURE 2:**
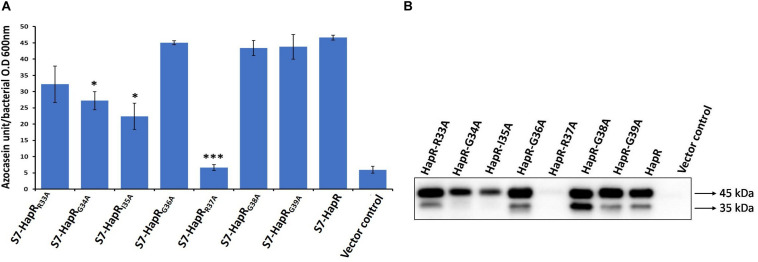
HA/P protease activity and production in recombinant derivatives of *Vibrio cholerae* strain S7. **(A)** Azocasein assay. The strains were grown for 12 h in TSB-D at 37°C (200 rpm) and the proteolytic activity was determined by assaying the digestion of azocasein. Values are the mean ± SD (*error bars*) plotted from experiments performed in triplicate (*p < 0.05, ***p < 0.001; unpaired two-tailed Student’s *t*-test). S7-HapR was taken as the positive control. **(B)** Western blot analysis of Hap production in the cell-free supernatants. The supernatants of the 12 h grown cultures were concentrated to 200 μl (10-fold) through 10 kDa Amicon ultracentrifugal filters (Millipore). Hap protein was detected by probing rabbit anti-Hap serum primary antibody and horseradish peroxidase (HRP)-conjugated goat anti-rabbit immunoglobulin G (IgG) secondary antibody. Molecular masses were deduced by referring to the protein molecular weight markers. The 45- and 35-kDa sizes of the protein denote the differently processed HAP protein.

In order to identify the critical residue(s) in HapR–DNA interaction, we replaced each residue of the glycine linker by alanine from positions 33 to 39 (^33^RGIGRGG^39^; Figure 1). As HapR acts as an activator and repressor, we have chosen two well-characterized HapR targets, namely, HapA (hemagglutinin/protease) and AphA, to evaluate the function of alanine variants. HapR induces protease (HapA) production at a high cell density (Zhu et al., 2002). To evaluate functionality, the alanine variants of the linker region were tested for their capacity to reinstate protease production in *V. cholerae* strain S7 harboring a non-functional HapR (Supplementary Table 1). Protease production was measured with cell-free supernatants of the recombinant strains containing various alanine derivatives by employing a chromogenic substrate assay published earlier (Benitez et al., 2001; Singh et al., 2014). Out of all the variants, only three strains, those harboring HapR-G34A, HapR-I35A, and HapR-R37A, showed a significant loss in protease activity as compared to the vector and functional HapR controls (Figure 2A). Of these, the strain expressing HapR-R37A had the most dramatic defect in protease activity. The protease result was further corroborated by Western blot of HapA protease (Figure 2B). Our data also indicated the presence of both 45 and 35 kDa matured processed forms, as evidenced earlier (Pal and Wai, 2013). It could be surmised that loss in the protease activity of HapR-R37A could be a result of the instability of the protein under *in vivo* condition. To rule out the possibility, FLAG epitope was inserted in the C-terminal variant of the wild-type and alanine variants of HapR and stability was examined by Western blot (Supplementary Figure 1). To ensure that FLAG insertion did not alter the activity of the wild-type and alanine variants of HapR, protease production was examined with FLAG-tagged constructs. We observed no significant alteration in protease production as compared to the non-FLAG-tagged constructs (Figure 2A and Supplementary Figure 2). Our Western blot data clearly indicated that HapR-R37A is stable and its intactness is comparable to other alanine variants and wild-type protein. In other words, the loss in activity of HapR-R37A is not linked to its *in vivo* stability.

To examine the generality of our observation and rule out any possible strain-specific phenomenon, all the alanine variants along with the vector and wild-type functional HapR were transformed in another protease-negative strain of *V. cholerae* V2_S_ (Dongre et al., 2011; Supplementary Table 1). We observed a similar trend of protease production in this strain background as well, where HapR-R37A exhibited a non-functional trait (Supplementary Figure 3). Taken together, the results obtained from protease with cell-free culture supernatants of *V. cholerae* strains S7 and V2_S_ confirmed the compromise in the functionality of the HapR-R37A variant.

**FIGURE 3:**
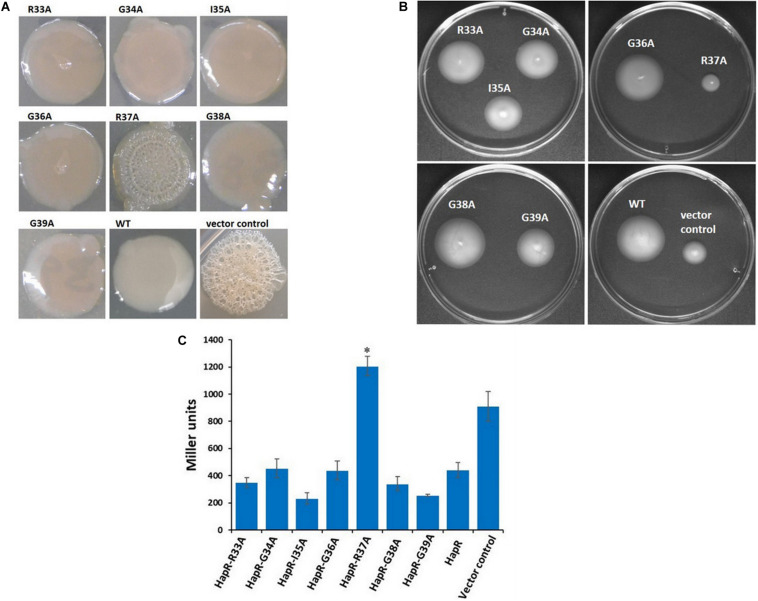
Colony morphology, motility phenotypes, and β-galactosidase reporter assay to examine the functionality of HapR and its alanine variants. **(A)** Colony corrugation was examined on Luria–Bertani (LB) agar containing appropriate antibiotics after incubation at 30°C for 48 h. **(B)** Motility phenotypes were analyzed on swarm plates after incubation at 37°C for 12 h. **(C)** The *Vibrio cholerae lacZ* reporter strain GK178 (*aphA-LacZ* fusion) and the recombinants were grown in AKI medium at 37°C for 12 h. All strains were diluted in fresh AKI media and allowed to grow up to 0.3–0.4 OD_60__0__*n*__*m*_. The β-gal production was measured as a readout of *o*-nitrophenol, a cleaved product of substrate ONPG, and further calculated the Miller unit. The amount of Miller units denoted the levels of *lacZ* expression and correlates the functionality of wild-type HapR and its alanine variants. HapR was taken as the positive control. Values shown are the mean and standard error of at least two biological experiments and three technical replicates (*p < 0.05; unpaired two-tailed Student’s *t*-test).

HapR negatively regulates *vps* gene expression and suppresses biofilm formation in *V. cholerae* (Beyhan et al., 2007; Lim et al., 2007). In some *V. cholerae* strains, mutation in HapR leads to the development of a rugose colony morphology (Yildiz et al., 2004). Interestingly, out of the two HapR-negative strains used in this study, *V. cholerae* V2_S_ produces rugose colony on LB agar (Figure 3A). Subsequently, all recombinant strains of *V. cholerae* V2_S_ carrying alanine variants along with the vector and wild-type functional HapR were examined for rugose colony development. We observed smooth colonies in all recombinant strains except the strain having HapR-R37A, which exhibited rugose morphotype (Figure 3A). As reported, rugose strains are less motile than their smooth counterparts (Ali et al., 2002). To examine this, motility assay was performed following a published protocol (Moisi et al., 2009). We observed that the smooth variants are more motile than the rugose variant (Figure 3B).

HapR represses the expression of the gene encoding AphA by binding to its promoter region and occludes the engagement of VpsR, the latter being an activator of AphA (Kovacikova and Skorupski, 2002; Lin et al., 2007). To examine the interaction of HapR-R37A with the promoter region of AphA, a *lacZ* reporter assay was conducted in a recombinant *V. cholerae* strain C6706, designated as GK178, carrying chromosomal fusion of *aphA–lacZ* (Supplementary Table 1; Kovacikova and Skorupski, 2002). In the reporter strain, binding of functional HapR to the *aphA* promoter region will block *lacZ* expression; therefore, the presence or absence of *lacZ* expression is directly proportional to the function of HapR. Our reporter assay with *V. cholerae* strain GK178 harboring all linker alanine variants and wild-type functional HapR clearly indicated that HapR-R37A failed to inhibit *lacZ* expression, further indicating its inability to bind the *aphA* promoter region (Figure 3C).

**FIGURE 4:**
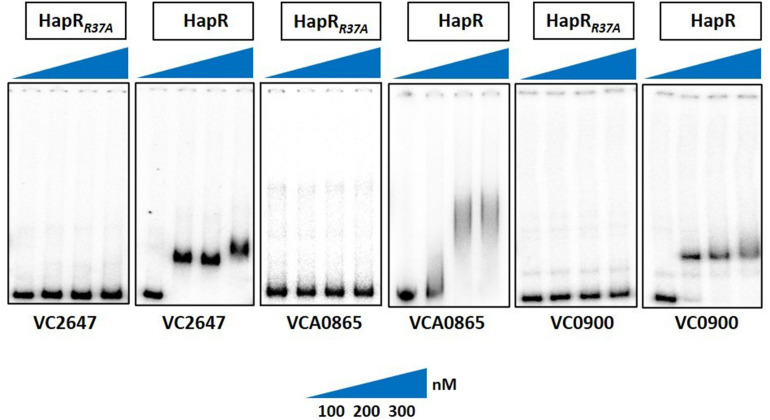
Gel shift experiments showing the inability of mutant HapR to bind to its cognate promoters. Electrophoretic mobility shift assay (EMSA) of the purified wild-type protein HapR and its mutant HapR_*R*__3__7A_ was performed with ^32^P-labeled promoter regions of *aphA* (VC2647), *hapA* (VCA0865), and *cdgG* (VC0900). The *solid wedges* represent the increasing concentrations of proteins. The results are representative of two or more experiments.

Since arginine at position 37 (R^37^) turned out to be the most important residue among others in the glycine linker, we subsequently kept our experimental focus on R^37^ to understand its role in the HapR regulatory activity. HapR directly interacts with the promoter region of *aph*A and *hap*A, thus modulating the expression of these proteins (Zhu et al., 2002). To evaluate the binding ability of HapR-R37A with these promoter regions, gel shift assays were performed as described earlier (Dongre et al., 2011). Gel shift analysis clearly demonstrated complete failure of the binding of HapR-R37A to the promoter region of *aphA* and *hap*A over a threefold range of concentrations (Figure 4). The data are in congruence with *in vivo* protease, rugosity, motility, and *lacZ* reporter assays (Figures 2A, 3), which further bolstered the importance of arginine 37 in the DNA binding regulatory activity of HapR.

As promoter regions of *aph*A and *hap*A harbor HapR binding motif 2, we chose another cognate promoter, *vc0900*, having HapR binding motif 1 (Tsou et al., 2009) and examined the DNA binding ability of HapR-R37A. Our data clearly demonstrated the inability of HapR-R37A to shift the promoter regions of *vc0900* (Figure 4).

In essence, combined *in vivo* and *in vitro* results aid in the identification of an essential arginine at position 37 (R^37^) for HapR function. Replacement of arginine with alanine (R37A) renders a completely non-functional HapR that fails to bind with the cognate promoters examined in this study.

### Necessity of Positive Charge at Position 37 to Maintain HapR Regulatory Function

**FIGURE 5:**
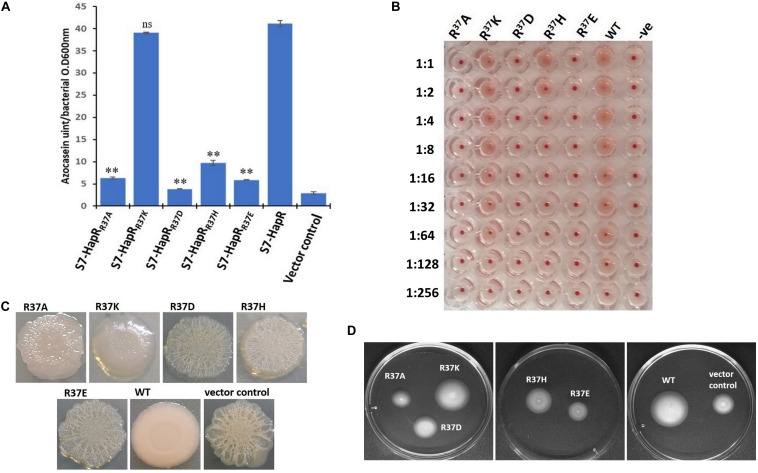
*In vivo* assays of the recombinant derivatives of *Vibrio cholerae* strains S7 and V2_S_ bearing wild-type HapR and its R^37^ variants (R37A, R37K, R37D, R37H, and R37E). **(A)** Protease activity. All *V. cholerae* S7 recombinant strains were grown for 12 h in TSB-D at 37°C (200 rpm) and the proteolytic activity was determined in cell-free culture supernatants by assaying the digestion of azocasein. Values are the mean ± standard deviation (*error bars*) plotted from experiments performed in triplicate (**p < 0.01; unpaired two-tailed Student’s *t*-test; *ns*, non-significant). S7-HapR was taken as the positive control. **(B)** Hemagglutination activity. The assay was performed by making a serial twofold dilution of the culture supernatants of the overnight (12 h)-grown *V. cholerae* S7 recombinant variants. The samples were incubated with 1.2% washed chicken erythrocytes and the ability of HA/P to hemagglutinate the red blood cells (RBCs) was checked. The results are representative of three independent experiments. **(C)** Colony morphology. Colony morphotype of *V. cholerae* V2_S_ recombinant strains was examined on Luria–Bertani (LB) agar containing appropriate antibiotics after incubation at 30°C for 48 h. **(D)** Motility phenotypes. Phenotypic analysis of the motility of *V. cholerae* V2_S_ recombinant variants was done on swarm plates containing appropriate antibiotics with 12 h post-incubation at 37°C.

**FIGURE 6:**
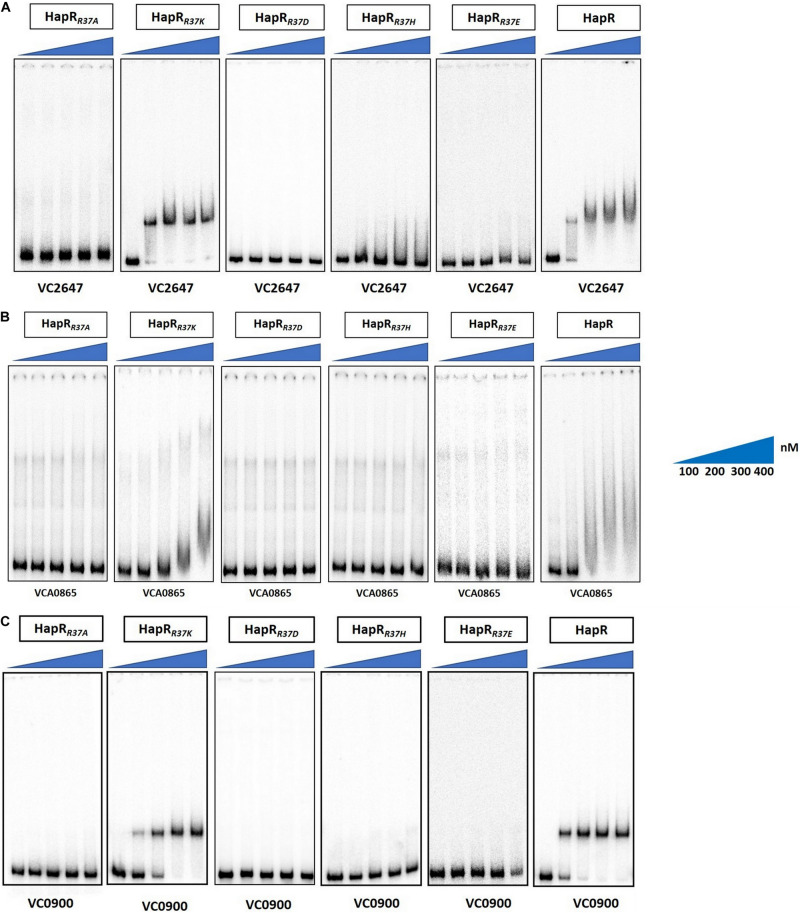
Gel shift experiments of HapR and its R^37^ mutants. Electrophoretic mobility shift assay (EMSA) of the purified wild-type protein HapR and its mutants HapR_*R*__3__7A_, HapR_*R*__3__7K_, HapR_*R*__3__7D_, HapR_*R*__3__7H_, and HapR_*R*__3__7E_ was performed with ^32^P-labeled promoter regions of **(A)** *aphA* (VC2647), **(B)** *hapA* (VCA0865), and **(C)** *cdgG* (VC0900). The *solid wedges* represent the increasing concentrations of proteins. The results are representative of two or more experiments.

It is evident from the previous section that arginine 37 is indispensable for HapR function. The question arises whether residue or charge is important at position 37. It should be noted that replacement of arginine with lysine is also associated with functional modulation, further indicating the importance of residue over charge (Aukerman et al., 1991; Tang et al., 2009). To address this, arginine 37 was substituted with lysine, glutamate, and aspartate. Incidentally, a natural variant of arginine 37 has been reported where the residue is substituted with histidine (Wang et al., 2011). All variants (R37A, R37K, R37E, R37D, and R37H) were subjected to a battery of *in vivo* and *in vitro* experiments (e.g., protease, hemagglutination, rugosity, motility, and gel shift). As documented, the HapR-R37K variant is functionally equivalent, while the functionality of the HapR-R37A, HapR-R37D, and HapR-R37E variants is significantly compromised in all experimental conditions in comparison to their wild-type counterpart (HapR-R37) (Figures 5, 6), whereas HapR-R37H appears to retain partial activity. To underscore that the loss in functionality of the HapR-R37D, HapR-R37E, and HapR-R37H variants is not linked with protein stability, FLAG Western blot was performed. No significant changes were observed in protease production after insertion of the FLAG epitope at the C-terminus of these variants (Supplementary Figure 4). Western blot data also documented the *in vivo* stability of all proteins (Supplementary Figure 5).

### DNA Binding Impairment Does Not Affect Oligomerization

**FIGURE 7:**
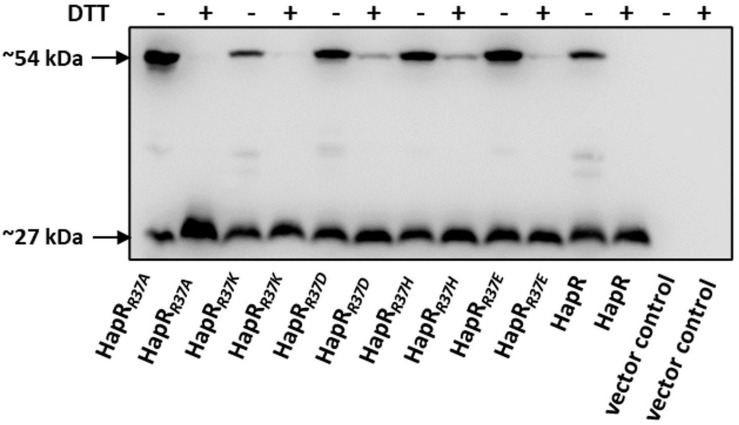
*In vivo* oligomerization. S7 strain of *Vibrio cholerae* possessing the C-terminal FLAG-tagged *hapR* and its R37 mutant derivatives were propagated in TSB-D until the late log phase. Equal numbers of cells were harvested and the samples were prepared from cell lysates in duplicate ± DTT (100 mM in the loading buffer). Samples were subjected to gel electrophoresis and the oligomeric state of the proteins was detected by immunoblotting with anti-FLAG antibody. The sizes ∼27 and 54 kDa depict the monomeric and dimeric states of the FLAG-tagged HapR protein, thereby suggesting that the mutations do not affect the oligomerization of the protein.

As documented, HapR exists as a dimer (De Silva et al., 2007; Dongre et al., 2011). It could be surmised that the non-functionality of the HapR-R37A, HapR-R37H, HapR37E, and HapR-R37D variants could be a result of an altered oligomeric pattern under *in vivo* condition. To further investigate, the oligomeric status under *in vivo* condition was examined by Western blot following protocols published earlier (Yang et al., 2013). Western blot data clearly exhibited the dimeric status of HapR-R37A, HapR-R37H, HapR-R37E, and HapR-R37D under the experimental condition (Figure 7).

### MD Simulation Unravels the Role of Arg37 (R^37^) in HapR-Mediated DNA Binding

**FIGURE 8:**
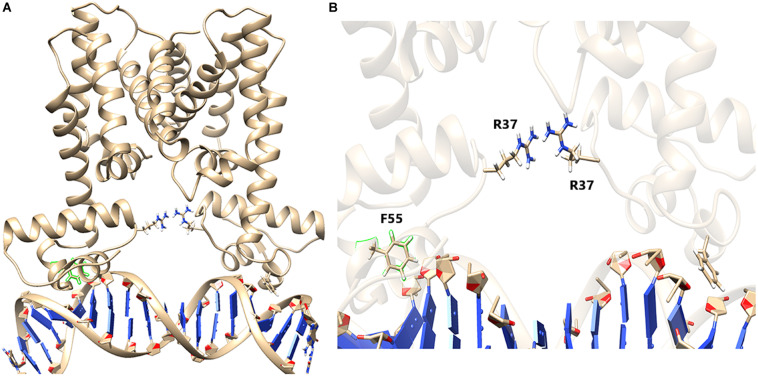
HapR with bound DNA. **(A)** HapR–DNA complex after 80 ns of non-restrained molecular dynamics (MD) simulation. Arginine 37 (R37) from both the chains are represented in a ball-and-stick model. **(B)** Close-up view shows that the orientation of R^37^ residues forms an ion pair possessing a “handshake” topology.

De Silva et al. (2007) demonstrated that Phe55 is very crucial for DNA binding by HapR. However, in this study, using various experimental approaches, we have been able to point out that, besides F55, another residue (R^37^) plays a very crucial role in DNA binding. To generate a more vivid picture of the scenario, we further performed structural analysis of binding using extensive MD simulations. Earlier, the promoter region of *hap*A was considered in MD simulation experiments (Singh et al., 2014). In this study too, we used the same DNA sequence and modeled a B-DNA using 3D-DART web server (van Dijk and Bonvin, 2009). Energy minimization of the DNA was performed using AMBER ff99bsc0 force field (Wang et al., 2004). Further, to generate a HapR–DNA complex, a docking procedure was carried out using AutoDock-4.2 package (Morris et al., 2009). Docking results were screened to obtain a complex structure with a total energy of −9.7 kcal/mol. The docked structure was then subjected to 50 ns of NVT and NPT equilibration using AMBER force field (Wang et al., 2004) with position restrains applied to the complex. The well-equilibrated structure was further introduced to 80 ns of non-restrained MD simulation. Analyzing the final structure, we found out that F55 from both the chains of HapR was taking part in stabilizing the bound DNA by forming electrostatic interactions (De Silva et al., 2007; Singh et al., 2014). We also found out that the R^37^ residues from both the chains of HapR are forming a hydrogen bond with a “handshake” topology to hold the two domains of the protein in a desired distance for binding with the DNA (Figure 8). Analysis of the trajectories of the simulation also showed that the handshake is not due to any charged ions, added to neutralize the counterion during simulations, lying nearby R^37^ (Supplementary Figure 6). R^37^ is also found to form electrostatic interaction with the main chain oxygen atom of I35, although this interaction is not true for both the R^37^ residues. However, to substantiate our claim, we further needed to mutate the R^37^ and performed extensive MD simulation. For this purpose, we took the final structure produced by an earlier simulation and substituted both the R^37^ with alanine using PyMOL package (DeLano, 2009). We used alanine substitution to completely eliminate any possibility of ionic interactions between two of these residues and, in turn, would provide us a way to check whether R^37^’s “handshake” topology was affecting the DNA binding or we just hit on an artifactual phenomenon.

**FIGURE 9:**
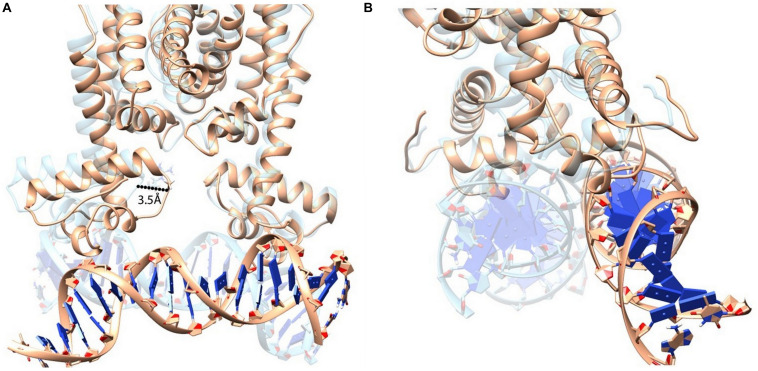
Superimposition of HapR-DNA bound states (HapR–DNA is represented in *light brown color*; HapR_*R*__3__7A_–DNA is represented in *transparent cyan color*). **(A)** Structural alignment shows that a large helix movement occurred due to the alanine substitution and which eventually led to the disruption of the protein–DNA complex. **(B)** 90° rotated view of the aligned structures.

The mutant (HapR-R37A) was then further equilibrated for 80 ns of NVT and NPT simulations until the backbone root mean square division (RMSD) reached a certain amount of stability (Supplementary Figure 7). The equilibrated structure was then further subjected to 80 ns of non-restrained MD simulation. Analysis of the trajectory showed that, after a few cycles of non-restrained simulations, the two domains of HapR-R37A failed to hold their position properly for providing interaction with the DNA. This vigorous domain movement eventually leads to the disruption of the electrostatic interactions between HapR-R37A and the DNA (Figure 9, Supplementary Figure 8, and Supplementary Video 1). Further, to check whether other positively charged amino acids have a similar effect, we chose to mutate the R^37^ with histidine (R37H). Simulations were carried out using a similar set of parameterizations to that used before. Non-restrained simulation results show that R37H is also unable to hold the DNA as the length of the histidine side chain is not long enough to establish the handshake (Supplementary Figure 9). In another similar simulation setup, we evaluated how the complex would behave if R^37^ is substituted with glutamate (E), which has the negatively charged side chains. The R37E simulations also failed to hold the DNA when subjected to 80 ns of non-restrained simulation. Analysis of the simulation snapshot shows that the *E* is 7 Å apart from each other and, due to this, is not able to form any kind of stabilizing interactions (Supplementary Figure 10). These MD simulation studies clarify the fact that the “handshake” mechanism of R^37^ is highly significant to fine-tune the domain movement in a corroborated manner, which allows the DNA to bind with HapR.

**FIGURE 10:**
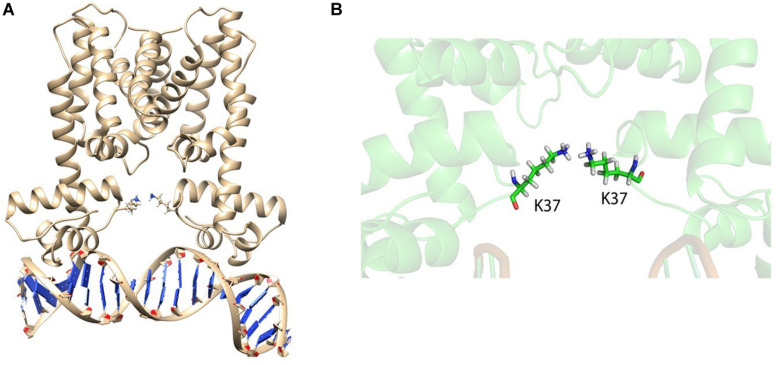
HapR_*R*__3__7K_ with bound DNA. **(A)** HapR_*R*__3__7K_–DNA complex after 80 ns of non-restrained molecular dynamics (MD) simulation. Lysine 37 (K37) from both the chains are represented as *sticks*. **(B)** Close-up snapshot of the K37 residues shows that these are forming ion pairs to hold the domains, controlling the rapid uncorrelated domain movement.

Based on our investigations performed so far, it could be hypothesized that not the residue in the position but the residue carrying specific charge matters establishes a positive HapR–DNA interaction. Hence, we further substituted the Arg37 residue with lysine, a residue closely resembling the same electrostatic properties. The mutant (HapR-R37K) was then equilibrated using 80 ns of NVT and NPT simulations to obtain a stable backbone RMSD (Supplementary Figure 11). After successful equilibration, another 80 ns of non-restrained MD simulation was carried out. Analysis of the trajectories of the non-restrained run of HapR-R37K showed that the rapid domain movements were very few compared to the HapR_*R*__3__7A_ mutant. Snapshots collected from the MD run showed that the Lys37 residue formed a hydrogen bond with each other and kept the vigorous domain movement at bay to facilitate the stability of the HapR (Figure 10), thus sustaining the HapR–DNA interaction. This also explains the fact that after 80 ns of non-restrained simulations, the DNA was found to be in a bound state with HapR-R37K. This phenomenon is also in good agreement with the *in vitro* and *in vivo* experimental study described in the preceding sections. Our current investigation delineates that the R^37^ residue is quite indispensable in HapR–DNA interactions, however, it can be replaced with specific residues with nearly similar electrostatic and structural signatures. In essence, our experimental and computational results have led to the identification of an arginine residue at position 37 that plays a critical role in the DNA binding activity of HapR. However, a similar investigation with the crystal structures of these complexes should provide more information if performed in the future.

## Discussion

HapR contributes immensely in host and non-host life cycles of *Vibrio cholerae* by modulating the function of the genes responsible for disparate cellular events in a cell density-dependent manner. To achieve this feat, HapR communicates effectively with a series of cognate promoters by recognizing the binding motifs in the promoter region and acting both as an activator and a repressor. The recognition is done by a putative DNA binding domain (DBD) situated in the N-terminal region spanning over three α-helices and comprising initial 57 amino acids, where helix 3 is the recognition helix. Previously, phenylalanine at position 55 (F^55^) in the recognition helix was the only amino acid linked to the DNA binding ability of HapR (De Silva et al., 2007). Later, the contribution of a glycine-rich linker connecting helices 1 and 2 in the DNA binding activity was brought to light (Dongre et al., 2011; Singh et al., 2014). The current study further delves into the composition of the linker region and eventually identifies an important arginine residue at position 37 contributing toward the DNA binding ability of HapR. The next question arises on the importance of charge vs. residue at this position. Interestingly, accumulated evidences indicating that a conserved arginine replaced with lysine impairs the DNA binding ability (Aukerman et al., 1991; Tang et al., 2009) left us to examine the effect of such substitution on R^37^ of HapR. Subsequently, R^37^ was replaced with negatively charged moieties such as aspartate and glutamate. We observed no loss in the DNA binding activity of the lysine-substituted variant, but the aspartate and glutamate variants turned functionally inert under experimental conditions. The data further reinforced the importance of positive charge rather than residue at position 37 for HapR–DNA cross talk.

It should be noted that natural variants of the glycine linker have also been reported where arginine 37 and glycine 39 were found to be substituted with histidine (R^37^H) and aspartate (G^39^D), respectively (Dongre et al., 2011; Wang et al., 2011). Previously, the molecular characterization of HapR-G39D has been done (Dongre et al., 2011; Cruite et al., 2018). In this study, we confirmed the non-functionality of R37H, described in the preceding sections.

The linker contributes immensely in the function of a protein. Though the majority of linkers are rigid, glycine-rich linkers are flexible in nature and maintain the functional integrity of discrete domains (Reddy Chichili et al., 2013). A large body of evidence bolsters the various functional roles of glycine-rich linkers in diverse proteins. While the glycine linker in PAX6, a human transcriptional factor, promotes contacts with DNA (Xu et al., 1999), the glycine linker in the transmembrane glycoprotein of retroviruses participates in membrane fusion (Wilson et al., 2005). Likewise, the glycine-rich linker of human T-cell leukemia virus type 1 transmembrane is also involved in membrane fusion (Wilson et al., 2005). In the case of SRSF1 (sequence-specific RNA binding factor), the glycine-rich linker connects two RNA recognition motifs and promotes interaction with splicing enhancers (Cho et al., 2011). Notably, mutations in the Gly-rich domain of TDP-43 have led to the development of amyotrophic lateral sclerosis (Pesiridis et al., 2009).

Quorum sensing defective strains of *V. cholerae* harboring non-functional variants of HapR are abundant in nature, and loss of HapR function also attributes certain physiological advantages to such strains (Joelsson et al., 2007; Dongre et al., 2011; Wang et al., 2011; Singh et al., 2013). Intriguingly, functionally challenged natural variants also aid in gaining novel mechanistic insights on HapR (Dongre et al., 2011; Singh et al., 2013). Incidentally, the present work stems out from our previous findings on the molecular basis of a non-functional natural quorum sensing variant where the significance of the glycine-rich linker, for the first time in the DNA binding activity of HapR, was revealed (Dongre et al., 2011). The current work further adds on the existing knowledge on HapR function by identifying a key arginine residue in linker sequence. It is noteworthy to mention that arginine 36 (equivalent to arginine 37 of HapR) of SmcR also contributes in DNA binding (Kim et al., 2010), further emphasizing the importance of arginine 37 in addition to phenylalanine 55 (De Silva et al., 2007) in the DNA binding activity of HapR. As both arginine 37 and phenylalanine 55 are well conserved among HapR homologs in several species of *Vibrio* (Supplementary Figure 12), it further necessitates evaluating the contribution of both residues in the functionality of HapR homologs in different *Vibrio* species. This warrants further investigation.

## Data Availability Statement

All datasets presented in this study are included in the article/Supplementary Material.

## Author Contributions

SR conceived the idea and wrote the manuscript. SR and ME designed the experiments and analyzed all biochemical data. ME performed the experiments. AM and SD performed and analyzed the MD simulation data. HS and RS contributed to the reagents, materials, and analysis tools. All authors gave editorial inputs.

## Conflict of Interest

The authors declare that the research was conducted in the absence of any commercial or financial relationships that could be construed as a potential conflict of interest.
