# Supplementary material for: Arginine 37 of Glycine Linker Dictates Regulatory Function of HapR
Source: Front Microbiol. 2020 Aug 21;11:1949. doi: 10.3389/fmicb.2020.01949 (PMC7472637; doi:10.3389/fmicb.2020.01949)
Supplement: Supplementary file 13 [file Table_1.docx]

| **Supplementary table 1.** Strains and plasmids used in this study  lemen | **Description** | **Source/reference** |
| --- | --- | --- |
| ***E. coli strains*** |  |  |
| Nova Blue | *E. coli* K-12, recA endA, lacIq, lacy, tet^r^ | Novagen |
| BL21(DE3) | *E. coli* B, F– ompT lon, with a λ prophage carrying the T7 RNA polymerase | Novagen |
| ***V. cholerae* strains** |  |  |
| V2 | Non-O1, non-O139, serogroup O37 | Ranjan K Nandy, National Institute of Cholera and Enteric Diseases (NICED), India |
| V2_S_ | Non-O1, non-O139, serogroup O37, hapR::pCD, Cm^r^ | Dongre et al., 2011 |
| V2_S_-vector control | V2_S_ having pKK177-3RI, Cm^r^ (17µg ml^-1^), Ap^r^ (100µg ml^-1^) | Dongre et al., 2011 |
| V2_S_-HapR | V2_S_ having HapR - pKK177-3RI,Cm^r^, Ap^r^ | Dongre et al., 2011 |
| V2_S_-HapR_R33A_ | V2_S_ having HapR_R33A_ - pKK177-3RI , Cm^r^, Ap^r^ | This study |
| V2_S_-HapR_G34A_ | V2_S_ having HapR_G34A_ - pKK177-3RI, Cm^r^, Ap^r^ | This study |
| V2_S_-HapR_I35A_ | V2_S_ having HapR_I35A_ - pKK177-3RI, Cm^r^, Ap^r^ | This study |
| V2_S_-HapR_G36A_ | V2_S_ having HapR_G36A_ - pKK177-3RI, Cm^r^, Ap^r^ | This study |
| V2_S_-HapR_R37A_ | V2_S_ having HapR_R37A_ - pKK177-3RI, Cm^r^, Ap^r^ | This study |
| V2_S_-HapR_G38A_ | V2_S_ having HapR_G38A_ - pKK177-3RI, Cm^r^, Ap^r^ | This study |
| V2_S_-HapR_G39A_ | V2_S_ having HapR_G39A_ - pKK177-3RI, Cm^r^, Ap^r^ | This study |
| S7 | Non-O1, non-O139, serogroup O37, *hapR* truncated with a stop codon at 118 position | Ranjan K Nandy, National Institute of Cholera and Enteric Diseases (NICED), India |
| S7-vector control | S7 having pKK177-3RI, Ap^r^ (100µg ml^-1^) | This study |
| S7-HapR | S7 having HapR-pKK177-3RI, Ap^r^ | This study |
| S7-HapR_R33A_ | S7 having HapR_R33A_-pKK177-3RI, Ap^r^ | This study |
| S7-HapR_G34A_ | S7 having HapR_G34A_-pKK177-3RI, Ap^r^ | This study |
| S7-HapR_I35A_ | S7 having HapR_I35A_-pKK177-3RI, Ap^r^ | This study |
| S7-HapR_G36A_ | S7 having HapR_G36A_-pKK177-3RI, Ap^r^ | This study |
| S7-HapR_R37A_ | S7 having HapR_R37A_-pKK177-3RI, Ap^r^ | This study |
| S7-HapR_G38A_ | S7 having HapR_G38A_-pKK177-3RI, Ap^r^ | This study |
| S7-HapR_G39A_ | S7 having HapR_G39A_-pKK177-3RI, Ap^r^ | This study |
| S7-HapR_R37K_ | S7 having HapR_R37K_-pKK177-3RI, Ap^r^ | This study |
| S7-HapR_R37D_ | S7 having HapR_R37D_-pKK177-3RI, Ap^r^ | This study |
| S7-HapR_R37H_ | S7 having HapR_R37H_-pKK177-3RI, Ap^r^ | This study |
| S7-HapR_R37E_ | S7 having HapR_R37E_-pKK177-3RI, Ap^r^ | This study |
| GK178 | C6706 str2 Δ*lacZ3, aphA-lacZ,* Sm^r^ (100µg ml^-1^) | [Kovacikova and Skorupski, 2002](https://onlinelibrary.wiley.com/doi/full/10.1111/j.1365-2958.2007.05693.x#b25) |
| GK178-vector control | GK178 carrying pKK177-3RI, Sm^r^, Ap^r^ | This study |
| GK178-HapR | GK178 carrying HapR-pKK177-3RI, Sm^r^, Ap^r^ | This study |
| GK178-HapR_R33A_ | GK178 carrying HapR_R33A_-pKK177-3RI, Sm^r^, Ap^r^ | This study |
| GK178-HapR_G34A_ | GK178 carrying HapR_G34A_-pKK177-3RI, Sm^r^, Ap^r^ | This study |
| GK178-HapR_I35A_ | GK178 carrying HapR_I35A_-pKK177-3RI, Sm^r^, Ap^r^ | This study |
| GK178-HapR_G36A_ | GK178 carrying HapR_G36A_-pKK177-3RI, Sm^r^, Ap^r^ | This study |
| GK178- HapR_R37A_ | GK178 carrying HapR_R37A_-pKK177-3RI, Sm^r^, Ap^r^ | This study |
| GK178-HapR_G38A_ | GK178 carrying HapR_G38A_-pKK177-3RI, Sm^r^, Ap^r^ | This study |
| GK178-HapR_G39A_ | GK178 carrying HapR_G39A_-pKK177-3RI, Sm^r^, Ap^r^ | This study |
| ***Plasmids*** |  |  |
| pKK177-3RI | Ap^r^ (100µg ml^-1^) | Giesla Stroz, National Institute of Health, U.S.A |
| pET15b | Ap^r^ (100µg ml^-1^), N-terminal 6 His-tag expression vector | Novagen |
| HapR-pKK177-3RI | 612 bp functional *hapR* V2G cloned in SmaI/HindIII site of pKK177-3RI Ap^r^ | Dongre et al., 2011 |
| HapR_R33A_-pKK177-3RI | *hapR* cloned in pKK177-3RI having R33 substituted with A, Ap^r^ | This study |
| HapR_G34A_-pKK177-3RI | *hapR* cloned in pKK177-3RI having G34 substituted with A, Ap^r^ | This study |
| HapR_I35A_-pKK177-3RI | *hapR* cloned in pKK177-3RI having I35 substituted with A, Ap^r^ | This study |
| HapR_G36A_-pKK177-3RI | *hapR* cloned in pKK177-3RI having G36 substituted with A, Ap^r^ | This study |
| HapR_R37A_-pKK177-3RI | *hapR* cloned in pKK177-3RI having R37 substituted with A, Ap^r^ | This study |
| HapR_G38A_-pKK177-3RI | *hapR* cloned in pKK177-3RI having G38 substituted with A, Ap^r^ | This study |
| HapR_G39A_-pKK177-3RI | *hapR* cloned in pKK177-3RI having G39 substituted with A, Ap^r^ | This study |
| HapR_R37K_-pKK177-3RI | *hapR* cloned in pKK177-3RI having R37 substituted with K, Ap^r^ | This study |
| HapR_R37D_-pKK177-3RI | *hapR* cloned in pKK177-3RI having R37 substituted with D, Ap^r^ | This study |
| HapR_R37H_-pKK177-3RI | *hapR* cloned in pKK177-3RI having R37 substituted with H, Ap^r^ | This study |
| HapR_R37E_-pKK177-3RI | *hapR* cloned in pKK177-3RI having R37 substituted with E, Ap^r^ | This study |
| HapR-pET15b | 612 bp functional *hapR* V2G cloned in NdeI/BamHI site of pET15b, Ap^r^ | Dongre et al., 2011 |
| HapR_R37A_-pET15b | *hapR* cloned in pET15b having R37 substituted with A, Ap^r^ | This study |
| HapR_R37K_-pET15b | *hapR* cloned in pET15b having R37 substituted with K, Ap^r^ | This study |
| HapR_R37D_-pET15b | *hapR* cloned in pET15b having R37 substituted with D, Ap^r^ | This study |
| HapR_R37H_-pET15b | *hapR* cloned in pET15b having R37 substituted with H, Ap^r^ | This study |
| HapR_R37E_-pET15b | *hapR* cloned in pET15b having R37 substituted with E, Ap^r^ | This study |
| HapR-FLAG-pkk177-3RI | *hapR* tagged with 3X FLAG at C-terminal end, cloned into SmaI/HindIII sites of pKK177-3RI. FLAG was amplified from the p3XFLAG-CMV-10 Expression Vector (Sigma Aldrich). | Singh et al., 2013 |
| HapR_R33A_-FLAG-pkk177-3RI | *hapR-*R33A tagged with 3X FLAG at C-terminal end, cloned into pKK177 3RI, Ap^r^ | This study |
| HapR_G34A_-FLAG-pkk177-3RI | *hapR-*G34A tagged with 3X FLAG at C-terminal end, cloned into pKK177 3RI, Ap^r^ | This study |
| HapR_I35A_-FLAG-pkk177-3RI | *hapR-*I35A tagged with 3X FLAG at C-terminal end, cloned into pKK177 3RI, Ap^r^ | This study |
| HapR_G36A_-FLAG-pkk177-3RI | *hapR-*G36A tagged with 3X FLAG at C-terminal end, cloned into pKK177 3RI, Ap^r^ | This study |
| HapR_R37A_-FLAG-pkk177-3RI | *hapR-*R37A tagged with 3X FLAG at C-terminal end, cloned into pKK177 3RI, Ap^r^ | This study |
| HapR_G38A_-FLAG-pkk177-3RI | *hapR-*G38A tagged with 3X FLAG at C-terminal end, cloned into pKK177 3RI, Ap^r^ | This study |
| HapR_G39A_-FLAG-pkk177-3RI | *hapR-*G39A tagged with 3X FLAG at C-terminal end, cloned into pKK177 3RI, Ap^r^ | This study |
| HapR_R37K_-FLAG-pkk177-3RI | *hapR-*R37K tagged with 3X FLAG at C-terminal end, cloned into pKK177 3RI, Ap^r^ | This study |
| HapR_R37D_-FLAG-pkk177-3RI | *hapR-*R37D tagged with 3X FLAG at C-terminal end, cloned into pKK177 3RI, Ap^r^ | This study |
| HapR_R37H_-FLAG-pkk177-3RI | *hapR-*R37H tagged with 3X FLAG at C-terminal end, cloned into pKK177 3RI, Ap^r^ | This study |
| HapR_R37E_-FLAG-pkk177-3RI | *hapR-*R37E tagged with 3X FLAG at C-terminal end, cloned into pKK177 3RI, Ap^r^ | This study |
