# Supplementary material for: Arginine 37 of Glycine Linker Dictates Regulatory Function of HapR
Source: Front Microbiol. 2020 Aug 21;11:1949. doi: 10.3389/fmicb.2020.01949 (PMC7472637; doi:10.3389/fmicb.2020.01949)
Supplement: Supplementary file 14 [file Table_2.docx]

**Supplementary table 2. Primers used in this study**

| **Name of primer** | **Primer sequence (5’- 3’)** |
| --- | --- |
| Sma I HapR | TAACCCGGGATGGACGCATCAATC |
| HindIII HapR | CCCAAGCTTCTAGTTCTTATAGATAC |
| NdeI HapR | GGGAATTGCATATGATGGACGCATCAATCGAAAAAC |
| BamHI HapR | CGCGGATCCCTAGTTCTTATAGATACACAG |
| pkk177-3RI Fwd | GCTCAAGGCGCACTCCCGTTTTG |
| Sma I flag Fwd | TCCCCCGGGATGGACTACAAAGAC |
| FLAG HindIII Rev | CCCAAGCTTTTACTTGTCATCGTCATC |
| HapR C-terminal FLAG OL Fwd | GTGTATCTATAAGAACGACTACAAAGACCATG |
| HapR C-terminal FLAG OL Rev | CATGGTCTTTGTAGTCGTTCTTATAGATACAC |
| HapR R33A Fwd | GAAGTGTTTGCTAAAGCCGGCATTGGTC |
| HapR R33A Rev | TTTAGCAAACACTTCCAACGCGATTTC |
| HapR G34A Fwd | TGTTTGCTAAACGCGCCATTGGTCGTG |
| HapR G34A Rev | CGCGTTTAGCAAACACTTCCAACG |
| HapR I35A Fwd | GTTTGCTAAACGCGGCGCTGGTCGTGGTGGT |
| HapR I35A Rev | GCCGCGTTTAGCAAACACTTCCAACG |
| HapR G36A Fwd | CTAAACGCGGCATTGCTCGTGGTGGTC |
| HapR G36A Rev | CAATGCCGCGTTTAGCAAACACTTC |
| HapR R37A Fwd | CGCGGCATTGGAGCTGGTGGTCACGCAG |
| HapR R37A Rev | CTGCGTGACCACCAGCTCCAATGCCGCG |
| HapR G38A Fwd | GCGGCATTGGTCCTGGTCACGCAGATATTGCG |
| HapR G38A Rev | CACGACCAATGCCGCGTTTAGCAAACACTTC |
| HapR G39A Fwd | GCAATTGGTCGTGGTGCTCACGCAGATATTGCC |
| HapR G39A Rev | CACCACGACCAATGCCGCGTTTAGCAAACACTT |
| YF13 Fwd (promoter *aphA*) | GATCGGAATTCGAATGCGCAATACTGGTTAAC |
| YF12 Rev (promoter *aphA*) | GATCGGGATCCGATAACGTGTGGTAATGACATG |
| VC0865 Fwd (promoter *hapA*) | GGAATTCGACTCATGGGGACTTGC |
| VC0865 Rev (promoter *hapA*) | GGAATTCTCAGTTGCCGCTCCGGCCA |
| VC0900 Fwd (promoter *cdgG*) | TGGTTTAGTCGAGAGCTACTGCCG |
| VC0900 Rev (promoter *cdgG*) | GAGTGAAGGCCAAAGTCATTG |
| HapR R37K Fwd | CTAAACGCGGCATTGGTAAAGGTGGTCACGCAGATA |
| HapR R37K Rev | TATCTGCGTGACCACCTTTACCAATGCCGCGTTTAG |
| HapR R37E Fwd | CGCGGCATTGGTGAGGGTGGTCACGCAG |
| HapR R37E Rev | CTGCGTGACCACCCTCACCAATGCCGCG |
| HapR R37H Fwd | GCTAAACGCGGCATTGGTCATGGTGGTCAGGCA |
| HapR R37H Rev | CTGCGTGACCACCATGACCAATGCCGCGTTTAG |
| HapR R37D Fwd | TAAACGCGGCATTGGTGATGGTGGTCAC |
| HapR R37D Rev | GTGACCACCATCACCAATGCCGCGTTTA |
