# Supplementary material for: Arginine 37 of Glycine Linker Dictates Regulatory Function of HapR
Source: Front Microbiol. 2020 Aug 21;11:1949. doi: 10.3389/fmicb.2020.01949 (PMC7472637; doi:10.3389/fmicb.2020.01949)
Supplement: Supplementary file 15 [file Data_Sheet_1.doc]

# Supplementary figure 1: Western blot analysis of FLAG-tagged HapR and its glycine rich linker (R^33^GIGRGG^39^) alanine variants. Experiment was executed with 12 hrs grown cultures. The whole cell lysates were subjected to 12% SDS-PAGE. Protein samples were electrophoretically separated and subsequently transferred onto a polyvinylidene difluoride (PVDF) membrane and the proteins were detected with monoclonal HRP-conjugated anti-FLAG antibody. Prestained PAGEmark™ Protein Markers (G. biosciences) were run along for reference. ̴ 27kDa show the size of the FLAG-tagged HapR protein.

**Supplementary figure 2: Protease activity of FLAG-tagged HapR and its glycine rich linker (R^33^GIGRGG^39^) alanine variants**. *V. cholerae* S7 bearing the wild type HapR and its alanine linker variants were analysed for protease production in cell-free culture supernatants. Hence ruling out the possibility of loss in activity post FLAG-tagging the variable constructs. The values are means ± standard deviation (error bars) taken from experiments done in triplicates *(**p*< 0.01; unpaired, two tailed student’s t-test). S7-HapR was taken as positive control.

**Supplementary figure 3: Protease activity of HapR and its glycine rich linker (R^33^GIGRGG^39^) alanine variants in *V. cholerae* V2_S_.** The proteolytic activity of concerned derivatives was checked in another *V. cholerae* protease negative background, strain V2_s_. The values are means ± standard deviation (error bars) taken from experiments done in triplicates (***p*< 0.01; unpaired, two tailed student’s t-test). V2_S_-HapR was taken as positive control.

**Supplementary figure 4: Protease activity of FLAG-tagged recombinant derivatives of *V. cholerae* S7 bearing wild type HapR and its R^37^ variants (R37A, R37K, R37D, R37H, R37E).** *V. cholerae* S7 bearing the wild type HapR and its R^37^ variants were analysed for protease activity in cell-free culture supernatants. Hence ruled out the possibility of loss in activity post FLAG-tagging the R^37^ variable constructs. The values are means ± standard deviation (error bars) taken from experiments done in triplicates (**p*<0.05; ***p*<0.01; unpaired, two tailed student’s t-test). S7-HapR was taken as positive control.

**Supplementary figure 5: Western blot analysis of FLAG-tagged wild type HapR and its R^37^ mutant derivatives (R37A, R37K, R37D, R37H and R37E)**. Experiment was performed with whole cell lysates of 12 hrs grown cultures. Protein samples were analysed on 12% SDS-PAGE and subsequently transferred to a polyvinylidene difluoride (PVDF) membrane. Proteins were detected with monoclonal HRP-conjugated anti-FLAG antibody. Prestained PAGEmark™ Protein Markers (G. biosciences) was run along for reference. ̴ 27kDa show the size of the FLAG-tagged HapR protein.

**Supplementary Figure 6:** Simulation trajectory snapshot analysis shows that no charged ions (positive or negative) reside near the R37 residue (represented in sticks) to induce any constrained conformation. One of the R37 is shown to form electrostatic interaction with main chain of I35 residue, however, other R37 remains distantly apart from forming any main chain interactions.

**Supplementary figure 7:** RMSD (root mean square deviation) of mutant (HapR-R37A)

**Supplementary Figure 8:** Double alanine substituted HapR (HapR_R37A_) failed to keep DNA in bound state after 80 ns of non-restrained simulation. Rapid domain movement leads to dissociation of DNA from the protein.

**Supplementary Figure 9: (A)** Structural alignment of HapR_R37_ (color: cyan) and HapR_R37H_

(color: brown) after 80 ns of non-restrained simulation. **(B)** Dislocation of DNA caused by the dislocation of the binding domains. **(C)** The H37 lies too far away to form the handshake compared to R37.

**Supplementary Figure 10: (A)** DNA got dissociated from protein as R37E mutant cannot stabilize the complex. **(B)** E37 residue is too far apart to form any kind of stabilizing interaction compared to R37 (Color: R37- cyan, E37- pink).

**Supplementary Figure 11:** RMSD (root mean square deviation) of mutant (HapR-R37K)

**Supplementary Figure 12: Multiple sequence alignment (MSA) of HapR and its homologs in different *Vibrio* species.** MSA of HapR homologs showing amino acid sequence (DNA binding domain) similarity and conservation of important Arginine residue in the linker interceding α helix 1 and α helix 2 of N-terminal DNA binding domain. A.s. – *Aliivibrio salmonicidia*; A.f. - *Aliivibrio fischeri*; P.m. - *Photobacterium marinum*; V.t. – *Vibrio tubiashii*; V.p. – *Vibrio parahaemolyticus*; V.c. – *Vibrio campbellii*; V.a. – *Vibrio anguillarum*; V.c. – *Vibrio cholerae*; V.m. – *Vibrio mimicus*)
